# Photodynamic Therapy Using Intense Pulse Light to Treat an HIV Patient With Perianal Squamous Cell Carcinoma: A Case Report

**DOI:** 10.7759/cureus.27679

**Published:** 2022-08-04

**Authors:** Melissa Jomsky, Christian Hailey Summa, Matthew B Zarraga, Michelle Demory Beckler

**Affiliations:** 1 Biomedical Sciences, Nova Southeastern University Dr. Kiran C. Patel College of Allopathic Medicine, Fort Lauderdale, USA; 2 Medicine, Nova Southeastern University Dr. Kiran C. Patel College of Osteopathic Medicine, Fort Lauderdale, USA; 3 General Surgery, Geisinger Medical Center, Wilkes Barre, USA; 4 Dermatology, Z-Roc Dermatology, Fort Lauderdale, USA; 5 Microbiology and Immunology, Nova Southeastern University Dr. Kiran C. Patel College of Allopathic Medicine, Fort Lauderdale, USA

**Keywords:** hpv, photodynamic therapy, warts, hiv, dermatology

## Abstract

Anogenital warts are considered one of the most common sexually transmitted infections caused by the human papillomavirus (HPV). One of the primary considerations with HPV is the virus’s high rate to develop into squamous cell carcinoma (SCC). SCC is one of the leading causes of skin cancer with a variety of treatment options. The gold standard of treatment for SCC is surgical excision. Complications may arise for those that are considered immunocompromised, or lack of efficacy may be taken into consideration for nonsurgical approaches. Herein, we suggest prompt diagnosis and treatment with photodynamic therapy (PDT) of HPV lesions to prevent disease progression and reoccurrence.

The patient is a 54-year-old male nonsmoker with a past medical history of squamous cell carcinoma and HIV and presented with a perianal rash. Additionally, he confirmed associated symptoms of itchiness, irritation, and pain. At the time of his appointment, his CD4 count was 121 cells/µL, and he stated he was compliant with his antiretroviral therapy. Based on history and physical examination, the patient was empirically treated with oral fluconazole and topical nystatin-triamcinolone for two weeks. At follow-up, symptomatology worsened, and a biopsy was performed. Squamous cell carcinoma in situ was confirmed histologically. A secondary bacterial skin infection developed at the biopsy site. At this point, PDT was recommended due to compromised tissue and further risk of infection.

At present, a gold standard of care for HPV infection does not exist. Prompt diagnosis and treatment of these lesions are important to recognize due to the high risk of the development of squamous cell carcinoma. Complications of secondary bacterial infections can arise with current treatment for squamous cell carcinoma, particularly in the immunocompromised. Non-surgical approaches for HPV have been less than desirable with higher recurrence rates of HPV lesions. Herein, we suggest the consideration of PDT treatment for HPV and SCC.

## Introduction

Human Papilloma Virus (HPV) is a viral infection with over 100 identified strains that can manifest with fibrous papules at various parts of the body. Anogenital warts also referred to as condyloma acuminate (CA), are considered one of the most common sexually transmitted infections in adults. In 2014, the National Center for Health Statistics showed that approximately 42.5% of the total population is infected with genital HPV [1]. HPV strains are classified into two categories, low risk, and high risk. Low-risk strains may manifest as an outbreak of warts, but warts do not typically progress into cancerous cells. High-risk strains have a higher chance of manifesting into skin cancers if the warts are persistent or left untreated. High-risk strains tend to occur more prevalently in susceptible populations like the immunocompromised.

Infection with Human Immunodeficiency Virus (HIV) and its associated treatments can suppress the immune system further, putting patients at a significantly higher risk of HPV compared to HIV-negative patients [2]. Treatment of HPV, particularly high-risk strains, in HIV-positive patients is extremely important given the risk of cancer initiation and progression associated with HPV infection. The chances of an HIV-infected patient developing anal squamous cell carcinoma is almost 13 times higher in those with a history of HPV compared to those who have no history of past HPV infections [3].

Currently, there is no gold standard of care for HPV, and treatment efficacy varies with each patient's specific circumstances. HPV treatment has a high probability of reoccurrence, which is estimated to be about 29% to 39% within the first year after clearance [4]. Common patient-administered treatments include topical options such as imiquimod, 5-fluorouracil, and trichloroacetic acid, which is an extended process that can last six weeks to months. In a study where patients were compliant with applying topical imiquimod 5% to genital warts for 16 weeks, only about 50% of the patients had complete clearance and 13% experienced a reoccurrence [5]. Physician-administered treatments include cryotherapy, electrodesiccation, and curettage (ED&C), and photodynamic therapy, which has an efficacy rate ranging from 70% to 96% [6]. Due to the high reoccurrence rate among HPV patients, most require a combination and series of various treatment modalities.

Squamous cell carcinoma (SCC) is the second most common skin cancer, and each year there are about 200,000 to 700,000 occurrences in the US [7]. SCC can be caused by mutations in squamous cells of the epithelium, and most commonly occurs externally on the surface of the skin. SCC varies in the depth of the tumor into the epithelium. Accordingly, superficial tumors are referred to as SCC in situ (SCCis) or Bowen’s Disease, and more aggressive or deeper tumors such as invasive SCC. The gold standard of treatment for any SCC is surgical excision. A non-surgical approach may be required due to the location of the carcinoma or a patient’s comorbidities. Non-invasive or minimally invasive treatment options include topical chemotherapeutic creams, cryotherapy, curettage and destruction, and photodynamic therapy. Cryotherapy and topical chemotherapeutic creams usually result in poor clearance rates and a high probability of reoccurrence [7].

The location of the SCC can also dictate the treatment course. For example, in our case presentation, skin cancer in the anogenital region may have more limited options due to the lack of excess tissue needed for surgical closure, higher susceptibility to infections because of the increased bacterial load present in the area, and poor wound healing because of the continuous friction in this area. In the last decade, the occurrence of skin cancers in the anal region has increased with 90% of anal cancer patients being histologically positive for SCC [6]. This statistic highlights the need for more desirable treatment options for anal SCC patients.

Photodynamic therapy (PDT) has recently become a more popular treatment modality for both HPV infections and SCCis. PDT involves applying a photosensitizer to the target area, placing the area under occlusion for a period of time to allow cellular uptake, then exposing the area to a light source. The goal of this treatment modality is that rapidly dividing cells, like cancer cells, will be selectively targeted to undergo apoptosis. In dermatology, the usage of PDT is currently FDA-approved for the treatment of actinic keratoses. Damaged cells like tumor cells, virally infected cells, precancerous cells, etc. uptake the photosensitizer at a more rapid rate than healthy cells, which makes this treatment more selective to target cells in contrast to chemotherapy, which is not as selective.

## Case presentation

A 54-year-old male presented with a rash in the perianal area that had been present for months. He endorsed associated symptoms of itchiness, irritation, and pain. He stated he has a past dermatological history of squamous cell carcinoma. He also noted he is HIV positive, and his CD4 count at the time of his appointment was 121 cells/µL, below the normal range of 490-1,790 cells/µL. Additionally, he had a history of removal of a “perianal bump,” but was unsure of the official diagnosis. The patient stated that he was taking Truvada, an antiretroviral therapy (ART). He was sexually active with one same-sex partner and declined ever smoking tobacco products.

Treatment and management

**Figure 1:**
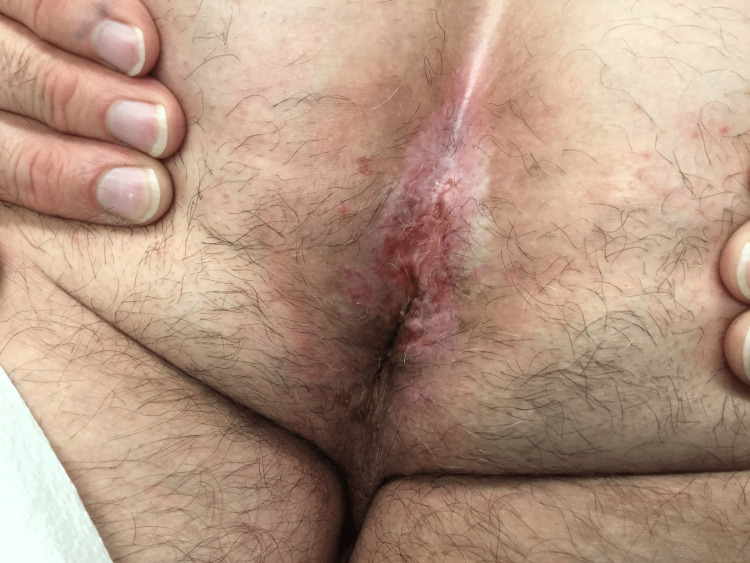
Before the first PDT treatment of the anus (June 2019) PDT- photodynamic therapy

The rash present in the perianal area appeared to be flaking and severely inflamed. He was clinically diagnosed with intertrigo and was empirically treated with oral fluconazole and topical nystatin-triamcinolone for two weeks. At his two-week follow-up, the patient’s rash did not improve, thus a biopsy was performed. The histological result of the biopsy was squamous cell carcinoma in situ. Six months later after no intervention due to the patient’s financial limitations, the affected tissue appeared to have spread extending past the original margins and involving more area and had increased in severity. The site appeared severely inflamed with open wounds and was clinically suspicious of a bacterial skin infection (Figure 1). At this point, PDT was recommended because the tissue was compromised, and the wounds elicited by PDT would be more minimal than ED&C and cryotherapy.

During his first treatment of PDT, the affected tissue was first cleansed with acetone, and then the photosensitizer, Ameluz® gel was applied. The gel was left on for 18 hours and then treated with an intense pulse light (IPL) laser, Cynosure® Palomar Icon laser, at 30 J/cm2, 30ms. The treatment had minimal efficacy, so a second PDT treatment was performed 3 weeks later. Before the application of Ameluz®, the site was debrided using a curette to allow the gel to penetrate deeper to increase the response rate. The next day, IPL was performed using the increased settings of 36 J/cm2, 30ms. Over the span of a few months, four consecutive ED&C procedures were performed with each treatment having significant improvement. After the fourth ED&C treatment, the base of the treated area was tested for residual cancerous cells and HPV infection, and it returned negative for both. The patient had follow-up visits at 13 and 24 months after his final treatment and was negative for any clinical reoccurrences.

## Discussion

To date, there is no generalizable data for the treatment of SCC progressed from chronic HPV infection in the perianal area of an HIV-positive patient using PDT. The factors that make this case unique are the use of Ameluz® as the photosensitizer, the use of IPL laser as the light source and the combination therapy of PDT and ED&C. These factors on their own may have yielded a different response, and could have various efficacy rates. However, the collective two treatments of Ameluz®-PDT with pre-procedure debridement, and four treatments of ED&C, most likely contributed to a 100% clearance rate as well as no reoccurrence to date.

PDT involves applying a photosensitizer to the target area, placing the area under occlusion for a period of time to allow cellular uptake, then exposing the area to a light source. The photosensitizer is a precursor in our cells to the hemoglobin synthesis pathway, and the external addition of the molecule creates an accumulation of protoporphyrin IX (PpIX). When exposed to a light source, PpIX spontaneously oxidizes into a free radical, and the build-up of these free radicals can result in cell death. Tumor cells absorb the exogenous photosensitizer more rapidly than healthy cells, which contributes to the specificity of this treatment for damaged cells without harming healthy cells.

PDT has two variables that can be manipulated based on the patient disease presentation: the photosensitizer and the light source. The current photosensitizers available are 5-aminolevulinic acid (ALA, Levulan®, Ameluz®) and methyl aminolevulinate (MAL, Metvix®), which are available in varying concentrations and can yield different results. Ameluz® ALA 10% gel was FDA approved in 2016 for the treatment of actinic keratoses on the face and scalp [8]. The nano-emulsified lipid technology allows ALA, which is a naturally hydrophilic compound, to penetrate deeper through lipophilic epithelial layers. A head-to-head study was performed to compare Ameluz® to MAL PDT treatments, and one treatment of Ameluz®-PDT had a 29.6% higher clearance rate than two treatments of MAL-PDT [8]. The Ameluz® gel allows for deeper penetration of lipophilic tissues comparatively to generic ALA, which would have been more effective for anal tissue, which is a semi-mucosal membrane. The recommended occlusion time for ALA is three to six hours [8], but the patient was occluded for 18 hours, which could have contributed to an increased accumulation of PpIX.

The other variable is the non-ionizing light source used, which varies based on availability in each clinic and can include LED lasers, intense pulsed light (IPL) devices, or lamps. The recommended spectrum for optimal activation of the photosensitizer is wavelengths of light between 600 and 800nm [9]. The light source used for this patient was the Cynosure ® Palomar Icon Max G, which is an IPL light device. LED lights are nearly monochromatic, so they only emit light waves within an extremely minimal range of wavelengths so it functions with the sole purpose of activating the photosensitizer. IPL is a polychromatic light source, so it delivers multiple light waves within two different spectrums of wavelengths. Each band of light emitted by the Max G IPL can serve different purposes, so the first pulse of 570-670nm is within the optimal window for photosensitizer activation. The second pulse of 870-1,200nm can target deeper vessels, which can cause vascular destruction and shut down the blood supply to the cancerous and virally infected cells. Disrupting blood and lymphatic vessels with ALA-PDT has shown significant results for long-term clearance and remission of HPV and SCC cells [5]. IPL also has the benefit of being able to manipulate the settings to alter the intensity of light dose delivered, unlike LEDs and lamps. The main side effect of PDT is pain, and being able to manipulate settings can also provide the benefit of pain control. If the patient is unable to tolerate the procedure, you can decrease the settings accordingly to mitigate the light intensity. Light devices can vary in pricing, and there are pros and cons for each light source. There is a lack of standardized delivery and dosimetry of PDT, because of the plethora of combinations of the variables possible. To date, there is no head-to-head studies comparing the efficacy of IPL vs LED light sources being used in PDT for the specific treatment of HPV or SCC.

HPV has become the most common skin infection in the HIV-infected since the development of anti-retroviral therapies. They may not be candidates for certain treatment modalities, like chemotherapeutic creams, that rely on the immune system for clearance. HIV patients have about a 32% clearance rate when using topical imiquimod, compared to the 50% clearance rate with non-HIV infected individuals [5,10].

Due to the patient’s immunocompromised status, other treatment modalities like surgical excision, MOHS, cryotherapy, etc., could leave HIV patients more susceptible to bacterial infections. Cutaneous infections like MRSA, and methicillin-resistant Staphylococcus aureus, are becoming increasingly concerning in the HIV community as a cause of morbidity and mortality [10]. In a study of MRSA infections, about one-third of 800 patients treated for MRSA were positive for HIV, and 41% had a reoccurrence of infection within four months of the initial presentation [9]. When treating an HIV patient, one of the factors to highly consider for the selection of treatment would be the severity of the wounds that are inflicted. Increasing the wound infliction will increase the risk of exposure to pathogens, and HIV patients are six times more likely to develop MRSA than non-HIV infected patients [10]. PDT would be a preferred treatment modality for an immunosuppressed patient because by altering the dosimetry you can control the intensity of wound infliction, unlike other modalities.

The patient’s CD4 count was 121 cells/µL, which demonstrates that the patient was compliant with his antiretroviral therapy (ART). PDT helps elicit an immune response by activating cellular cascades and has been shown to cause the upregulation of cytokines, dendritic cells, and memory cells [7]. In a patient with a low CD4 count, this is a desirable outcome that would facilitate the immune response in clearing the tumor cells and viral infection.

At the time of the first treatment, the integrity of the skin was too compromised to withstand an invasive treatment, because it would have left the patient with a severe wound and susceptible to a bacterial infection. Additional to the patient’s low CD4 count, the physician opted for a more non-invasive treatment modality initially. The first two treatments of PDT helped gain improvement of the area without being aggressive, which contributed to ED&C being a treatment option later down the line.

## Conclusions

Currently, there is no gold standard of care for HPV. Treatment of these lesions is important to take into consideration due to the high propensity for HPV to develop into SCC. Current treatments provide a vast range of efficacy, including both non-surgical and surgical approaches. Of note, HPV has become one of the most common skin infections in HIV patients. Due to the immunocompromised status of this patient population, common treatment modalities including cryotherapy and surgical excision have a higher rate of secondary bacterial infections. We acknowledge that there has been FDA approved for the treatment of actinic keratosis as well as the increasing popularity of PDT in HPV patients. However, unique to this case, we present an HIV/HPV-positive patient, with a poor CD4 count. Additionally, we are suggesting the consideration of PDT as a standard of therapy for these patients due to the decreased risk for infection and benefit of selectivity. Our case highlights a positive outcome of PDT treatment of an HIV/HPV-positive patient with complete clearance of margins and recurrences.
